# Determinants of physician empathy during medical education: hypothetical conclusions from an exploratory qualitative survey of practicing physicians

**DOI:** 10.1186/1472-6920-14-122

**Published:** 2014-06-22

**Authors:** Florian Ahrweiler, Melanie Neumann, Hadass Goldblatt, Eckhart G Hahn, Christian Scheffer

**Affiliations:** 1 Integrated Curriculum for Anthroposophic Medicine, Institute for Integrative Medicine, School of Medicine, Faculty of Health, Witten/Herdecke University, Alfred-Herrhausen-Straße 50, 58448 Witten, Germany; 2 Medizinische Klinik, Augusta-Kranken-Anstalt, Bergstraße 26, 44791 Bochum, Germany; 3 Faculty of Health, Witten/Herdecke University, Alfred-Herrhausen-Straße 50, 58448 Witten, Germany; 4 Department of Nursing, Faculty of Social Welfare & Health Sciences, University of Haifa, Mount Carmel, 3498838 Haifa, Israel; 5 Gesellschaft für Berufliche Fortbildung, Forschung und Entwicklung an der Medizinischen Klinik 1, Universitätsklinikum Erlangen, Ulmenweg 18, 91054 Erlangen, Germany; 6 Center for Educational Research, School of Medicine, Faculty of Health, Witten/Herdecke University, Alfred-Herrhausen-Straße 50, 58448 Witten, Germany

**Keywords:** Empathy, Medical education, Physician, Physician-patient relationship, Health communication, Promoting factors, Inhibiting factors

## Abstract

**Background:**

Empathy is an outcome-relevant physician characteristic and thus a crucial component of high-quality communication in health care. However, the factors that promote and inhibit the development of empathy during medical education have not been extensively researched. Also, currently there is no explicit research on the perspective of practicing physicians on the subject. Therefore the aim of our study was to explore physicians’ views of the positive and negative influences on the development of empathy during their medical education, as well as in their everyday work as physicians.

**Method:**

We administered a written Qualitative Short Survey to 63 physicians in seven specialties. They were able to respond anonymously. Our open-ended question was: “What educational content in the course of your studies and/or your specialist training had a positive or negative effect on your empathy?” We analyzed the data using thematic content analysis following Mayring’s approach.

**Results:**

Forty-two physicians took part in our survey. All together, they mentioned 68 specific factors (37 positive, 29 negative, 2 neutral) from which six themes emerged: 1. In general, medical education does not promote the development of empathy. 2. Recognizing the psycho-social dimensions of care fosters empathy. 3. Interactions with patients in medical practice promote empathy. 4. Physicians’ active self-development through reflective practice helps the development of empathy. 5. Interactions with colleagues can both promote and inhibit empathy through their role modeling of empathic and non-empathic behavior. 6. Stress, time pressure, and adverse working conditions are detrimental to empathy development.

**Conclusions:**

Our results provide an overview of what might influence the development of clinical empathy, as well as hypothetical conclusions about how to promote it. Reflective practice seems to be lacking in current medical curricula and could be incorporated. Raising physicians’ awareness of the psycho-social dimension of disease, and of the impact of peer influence and role modeling, seems promising in this regard, too. Stress and well-being seem to be closely related to physician empathy, and their modulation must take into account individual, social, and organizational factors. Further research should investigate whether or how these hypothetical conclusions can deepen our understanding of the determinants of physician empathy in order to help its promotion.

## Background

“Empathy is considered a basic component of all helpful relationships” ([1], p. S9), being “one of the strongest [motivations]” for pro-social behavior ([2], p. 320). It is a vital component of high-quality health care and an important aspect of medical practice and professionalism [1,3-8]. Several studies have shown that “clinical empathy and related physician behaviors towards their patients are associated with multiple positive effects” [5], e.g.: improved metabolic status in diabetic patients [9]; shorter duration and less severe course of the common cold [10]; improved physical and psychological health [11-14]; better compliance and satisfaction of patients [14,15]; enhanced patient enablement and coping [12,13,16,17]; better information exchange between physician and patient [11,15]; improved ease and accuracy of diagnosis [5]; better clinical performance of students and physicians [18-21]; and possibly more efficient resource utilization [22]. Accordingly, medical education associations and other professional organizations in several countries agree that empathy is a desirable physician characteristic that should be developed and promoted in the medical education process [23-28].

In contrast to the aim of fostering empathic interaction with patients, a recent systematic review by two of this article’s authors and other colleagues has described a statistically significant decline in the self-assessed empathy of medical students and residents in 16 of 18 studies from 1990 to 2010 [29]. They identified two main factors that significantly influenced the decline in empathy: 

• entering the clinical practice phase, including patient contact, and

• stress on the part of students and residents.

While changes in empathy during medical education and the effects and components of physician empathy have been reviewed and described in detail in earlier research [5,29], there is no such clear description of the factors that influence empathy development in a positive or negative way [29-31]. So the precise mechanisms that cause factors like those found by Neumann and colleagues [29] to have an effect remain unknown. An understanding of the determinants of empathy, however, is necessary in order to design “targeted” and evidence-based interventions for its promotion [29,32]. A number of researchers have investigated medical students’ views about the factors that influenced the development of empathy and compassion during their medical education [33-37]. However, our narrative MEDLINE search conducted via MEDPILOT (http://www.medpilot.de), last updated on September 19, 2013, and using the syntax (empathy OR compassion OR sympathy) AND (medical AND education) AND (doctor* OR physician* OR student*) AND (opinion* OR view* OR perspective* OR (empirical AND research) OR (data AND collection) OR (qualitative AND research)), did not yield any result for explicit research on the perspective of practicing physicians.

Because there was no systematic research on the physician-specific determinants of clinical empathy, we designed this study to explore the following question: 

What do physicians in different specialties experience as promoting and inhibiting the development of empathy during postgraduate training and in their memories of undergraduate medical education?

An analysis of physicians’ understanding of empathy, their perceptions of how empathy affects health outcomes, and their identification of barriers to empathic behavior in medical practice was recently published by two of this article’s authors and other colleagues, using additional data from the present study [38]. They identified barriers in the workplace and organizational environment, patient characteristics—e.g., “difficult patients”—, and personal attitudes and behaviors of the physicians. The main focus of the present study, however, is the influence of medical education on the promotion and inhibition of empathy. A secondary study aim was to investigate whether physicians’ experiences differed from specialty to specialty.

There has been much debate on the nature of empathy in the medical context [31,39]. In this study, we use the definition by Mercer and Reynolds: “Empathy involves an ability … (a) [to] understand the patient’s situation, perspective and feelings (and their attached meanings); (b) to communicate that understanding and check its accuracy; and, (c) to act on that understanding with the patient in a helpful (therapeutic) way” [1]. Empathy can include the affective experience of another person’s feelings *as if* they were one’s own. However, an empathic response always includes disentangling one’s own emotions from those of the other person. Sympathy, in contrast, does not entail an emotional separation from the other person’s emotional state and is therefore thought to interfere with clinical objectivity [5].

## Method

### Questionnaire

To collect the data, we used the Qualitative Short Survey, a short questionnaire with open-ended questions [38,40]. The use of open-ended questions in a survey is suitable for first insights into a little-researched field [40,41]. We used this tool because studies of physicians’ subjective views about the factors that promote or inhibit empathy are scarce, so there were no suitable instruments already developed [38,41]. Furthermore, the questionnaire allows for anonymity, and respondents who are assured of anonymity are less likely to give socially desirable responses [42]. Finally, because responding to our questionnaire required less time than an in-depth qualitative interview, it was better suited to physicians’ heavy and tightly scheduled workloads [38].

The questionnaire consisted of four pages, including one cover page, five open-ended questions on physician empathy, a few socio-demographic questions, and one question using a Likert-Scale on the assumed effects of empathy on patients’ health outcomes. For this article, we analyzed the responses to the following question: 

“What educational content in the course of your studies and/or your specialist training had a positive or negative effect on your empathy?”

The analysis of three other questions from the same study has recently been published elsewhere [38], and the complete questionnaire is included in the Additional file 1: Appendix.

### Sampling and data collection

In order to receive a variety of responses that would allow for comparisons between different types of physicians, we used a stratified purposeful sampling approach ([41], pp. 230–243) to survey physicians in internal medicine, general medicine, pediatrics and rehabilitation medicine. We assumed that more than four respondents per specialty would constitute a sufficient sample size for our exploratory study.

We contacted 15 physicians in each of the four specialties. Those members of our research group who were physicians themselves asked colleagues they knew personally in their respective professional associations to participate in the study. In addition, we asked one physician from neurology, one from psychosomatic medicine, and one from surgery to participate in the survey.

The data were collected from the beginning of March until mid-April 2010. We e-mailed the questionnaire or sent it by mail to the 63 physicians. All of the physicians received two reminders to complete the survey. The participating physicians could return the questionnaire by mail with a post-paid return envelope, by fax, or by e-mail.

### Sample description

All together, 42 of the 63 physicians contacted returned the questionnaire (a 67% response rate). Most of the respondents worked in an in-patient institution. Their socio-demographic data are provided in Table 1.

**Table 1:** Socio-demographic characteristics of the 42 participating physicians

	**Characteristics**	**Median**	**Range**	**Number**	**Percent**
Seniority	Age	45.5	25–67		
	Years of professional experience	14.5	0–38		
Gender	Men			31	73.8
	Women			10	23.8
	No details			1	2.4
Fields	Internal medicine			14	33.3
	Pediatrics			11	26.2
	Rehabilitation medicine			6	14.3
	General medicine			5	11.9
	Neurology			1	2.4
	Psychosomatic medicine and psychotherapy			1	2.4
	Surgery			1	2.4
	No details			3	7.1
Specialist qualification				29	69.0

### Data analysis

**Figure 1:**
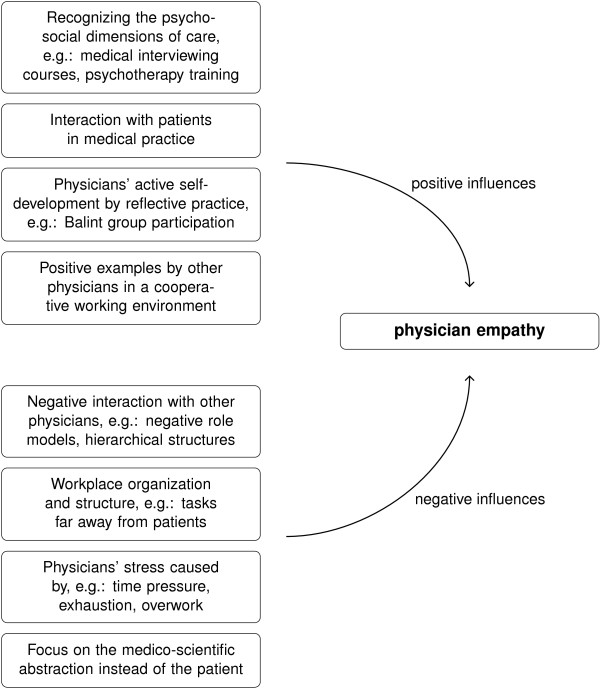
**Main themes with examples of the factors perceived to influence our respondents’ empathy.** Further details can be found in the Results and Discussion section.

The responses were transcribed and electronically stored as Word files [43]. After that, the primary analysis was done using a low-technology technique [44] with printouts, scissors, and crayons. We then used XMind mind-mapping software [45] to develop the coding scheme (included in the Additional file 1: Appendix) and TeX PGF/TikZ graphics software [46,47] to compile the main results (Figure 1). All quantitative analysis of the coding was done manually or with a calculator. The socio-demographic data of the respondents were analyzed using LibreOffice Calc [48].

To conduct the analysis, we used thematic content analysis, following Mayring’s inductive approach [49]. Where appropriate, we paraphrased and generalized respondents’ statements. We organized identical and similar passages by topic in order to identify individual influential factors, and then combined similar factors into major categories, to identify the main themes. Thus, we preserved the original content while producing an inductive, emergent summary at a higher level of abstraction. The resulting coding scheme, which is available in the Additional file 1: Appendix, was again applied to all of the material in order to verify its validity. After identifying the main themes, we grouped the factors associated with each theme according to whether the physician respondents believed the factor had had a positive or negative influence on the development of empathy, or was neutral.

Three of us (FA, CS, and MN) were involved in the analysis. First, we analyzed the transcripts independently (with each analyst using his or her coding scheme) and identified the main themes and specific factors mentioned by the respondents. In the next step we compared the three coding schemes, debated differences of opinion, and reached consensus on one scheme.

### Ethics statement

The data collection was completely anonymous (in accordance with the German Federal Data Protection Act), and participation was voluntary. All participants were considered to have full control over the extent to which they wanted to participate and to disclose personal data. Current research practice and legislation in Germany do not require an ethical approval process to conduct such a study [50].

## Results

### General description of responses and major themes

The physicians’ responses varied in form and length. Some provided lists of one-word responses, some answered in narrative phrases or whole sentences, and others used both. They included 1 to 7 specific factors per answer, and there were no missing responses to the question we are analyzing here. Sometimes, participants used punctuation or underlined words for emphasis.

All respondents either listed specific factors that they perceived to have had an impact on their empathy with patients, or provided statements about the general influence of medical education. Some did both. All together, we identified 66 factors with either a positive or negative influence on the physicians’ development of empathy in their practice of medicine: 37 factors could be labeled “positive”, 29 “negative”. The remaining 2 we labeled “neutral”. Positive factors were mentioned 112 times; negative factors were mentioned 73 times. Table 2 shows the 13 most frequently mentioned factors. The Additional file 1: Appendix contains the complete coding scheme with all factors listed in their respective major categories.

**Table 2:** The 13 most frequently mentioned positive (+) and negative (-) factors

**Factor**	**Times**	**+/-**
	**mentioned**	
Positive example of other physicians	15	+
Pursuing extracurricular activities on one’s	12	+
own initiative		
Lack of time and time pressure	9	-
Negative example of other physicians	8	-
Practice-based training	6	+
Training in psychology, psychiatry,	6	+
psychosomatic medicine, psychotherapy		
Training in interview and history taking	5	+
Non-medical experiences, studies, and	5	+
lectures		
Focus on scientific facts and guidelines in	5	-
teaching and practice		
Professional contact with patients in general	4	+
Good teachers and lectures	4	+
Complementary and alternative medicine	4	+
Bureaucracy and patient-remote tasks	4	-

Six main themes resulted from our analysis: 

1. In general, medical education does not promote the development of empathy.

2. Recognizing the psycho-social dimensions of care fosters empathy.

3. Interactions with patients in medical practice promote empathy.

4. Physicians’ active self-development through reflective practice helps the development of empathy.

5. Interactions with colleagues can both promote and inhibit empathy through their role modeling of empathic and non-empathic behavior.

6. Stress, time pressure, and adverse working conditions are detrimental to empathy development.

Although we explicitly asked the respondents to think about the content of their medical education, only themes 1 through 3 responded to our question, and correspondingly only 30 of the 68 specific factors were directly related to formal elements of the respondents’ education. The majority of factors relating to the other three themes listed above were not associated with the respondents’ medical education.

In considering whether the response patterns were associated with gender or medical specialty, we observed some trends: Women mentioned working conditions more often than men; pediatricians made more statements about physicians’ active self-development; and internists and general practitioners talked more about interactions with other physicians. The share of statements referring to themes 1, 2, or 3 was similar among all specialties (35–49%).

In the following subsections we provide more details about the positive and negative influence on respondents’ development of empathy.

### In general, medical education does not promote the development of empathy

Many respondents said that their empathy was little affected by their medical training. By and large, their statements give the impression that medical under- and postgraduate education was not helpful in the development of empathy. 

“[The development of empathy was] exclusively (almost) my own initiative, learning by doing” (internal medicine, male)

“There were no elements of medical education or specialist training that considered empathy at all.” (no details given)

A few respondents explicitly stated that their medical studies had *not* negatively influenced their development of empathy, for example: 

“There were neither explicit nor implicit elements of training which taught me that empathy was not desirable.” (internal medicine, male)

Maybe for this reason one respondent wondered whether empathy could be taught in medical school or if a person is naturally endowed with it. Another said that empathy is an individual skill that can be taught.

### Recognizing the psycho-social dimensions of care fosters empathy

Among the factors that the respondents said fostered physician empathy were specific curricular elements of medical education. These had in common that they focused on patient-physician interaction and/or the psycho-social characteristics of a patient. For example, respondents mentioned training in psychology, psychosomatic medicine, or psychotherapy, medical interviewing courses, the subject medical ethics, and attentiveness training. 

“Training in interviewing!!!” (general medicine, female)

“training in psychiatry and psychotherapy” (neurology and psychiatry, female)

“training in the subjects of medical ethics, psychology” (surgery, male)

“In the course of my studies, I took part in ‘attentiveness training’, which also covered the theme of projection; this opened my mind to it”. (internal medicine, female)

Additional areas mentioned were general practice, palliative care, complementary and alternative medicine, and salutogenesis.

In contrast to patient-centered educational elements, which most respondents considered valuable in fostering empathy, a few physicians mentioned that focusing on scientific facts, diagnostic results, and guidelines negatively influenced their development of empathy. 

“I find the numerous guidelines and ‘checking them off’ counterproductive (the internal agenda is guideline- rather than patient-oriented).” (general medicine, male)

“Suffering/pain were reduced to scientific phenomena, to be treated pharmacologically.” (pediatrics, male)

### Interactions with patients in medical practice promote empathy

Another group of factors centered on medical practice and, during undergraduate education, practice-based learning with patient contact. These two factors were perceived as helpful in developing clinical empathy. Respondents described this relationship in different ways and noted, for example, clerkships as medical students or daily practice as physicians. Also, interdisciplinary cooperation and constructive communication with patients were perceived to foster physician empathy. 

“during training … what the patient wanted to tell” (pediatrics, male)

“critical feedback from patients” (pediatrics, female)

One participant also emphasized that patient contact on a personal level was a positive factor, while two physicians said that patient contact was a barrier to the development of empathy. One said that his intensive care training required maintaining distance from patients in order to remain functional; the other had experienced disappointment when patients neglected her level of commitment to the patient’s well-being.

### Physicians’ active self-development through reflective practice helps the development of empathy

This theme comprises the extracurricular activities of respondents which they reported improved their empathy. Because these activities were not required by the formally endorsed medical curriculum, respondents took part on their own initiative. One respondent even emphasized that his empathy development was “exclusively (almost) …[his] own initiative” (internal medicine, male). These extracurricular activities were characterized by reflection and active self-development. For example, some reported conducting active reflection on biographies and psycho-social conditions of patients, or on the interactions they observed between colleagues and patients. Others spoke of both reflection and active self-development, such as Balint group participation and discussing end-of-life questions as part of a working group. 

“self-organized student history-taking group[;] student self-examination course[;] interviewing course in psychosomatic medicine[;] Balint group[;] encounter group in child and adolescent psychiatric training[;] … project leader of the children’s hospital working team in the death and dying project at [name of hospital]” (pediatrics, male)

“being supervised, and development of my own personality” (neurology and psychiatry, female)

Respondents also mentioned other non-medical private, academic, and religious experiences as supporting the development of empathy, such as “encounters with experienced, wise individuals” (pediatrics, male) and “general studies, attending lectures on other subjects, such as psychology” (no details). One respondent stated: 

“My empathy is more closely connected to my Christian perception of human beings.” (rehabilitation medicine, male)

### Interactions with colleagues can both promote and inhibit empathy through their role modeling of empathic and non-empathic behavior

In this theme we summarized the emergent impact on physician empathy from interactions with colleagues, teachers, and superiors.

Physicians viewed colleagues who demonstrated empathic and considerate behavior towards patients as role models with a positive influence. Colleagues who did not demonstrate such behavior, or who were cynical and detached were perceived as negative role models. The descriptions of role modeling seemed especially vivid. 

“experienced colleagues who cultivate an empathic manner with patients” (pediatrics, male)

“During the studies and training, we were frequently instructed: ‘Try to put yourself in the patient’s situation”’. (rehabilitation medicine, male)

“Examples of empathy were … provided by colleagues/superiors, who set both positive and negative examples.” (rehabilitation medicine, male)

“rather, many other colleagues (e.g., also physicians practicing orthodox medicine in an academic setting) who were not responsive to their patients and their problems (keyword: physicians’ arrogance, ‘gods in white’)” (internal medicine, male)

“cynicism and [physicians who saw] patients as fodder for [clinical] trials” (general medicine, male)

The respondents also mentioned other ways in which physicians influenced each other. Professional exchanges, learning from one another and a non-hierarchical and cooperative interdisciplinary working style were perceived as positive. In contrast, “hierarchical structures instead of teamwork on eye level” (internal medicine, male) were viewed negatively. In addition, physicians valued expertise and high-quality teaching as helping them practice empathy, e.g., good clinical teachers and exemplary lectures. Correspondingly, “bad instructors” (internal medicine, male) were not viewed as helpful.

### Stress, time pressure, and adverse working conditions are detrimental to empathy development

A group of adverse conditions for the development of physician empathy centered on stress at the workplace or during medical school. Respondents reported that pressure to perform, competition, “cramming for exams” (pediatrics, male), being overworked, exhaustion, or simply “the general stress in hospital” (pediatrics, male) all had a negative impact on their empathy or its development.

Additionally, in their comments on working conditions, nine of our 42 respondents mentioned lack of time and time pressure as inhibiting empathetic behavior. Sometimes, the time factor was mentioned in relation to other working conditions, thus making a connection between environmental factors and the respondent’s internal condition: 

“Frequently, because of *time pressure* [italics were simply underlined in the original] and the number of patients, [it is] not possible to be empathic towards parents and patients.” (pediatrics, male)

“Because of time pressure caused by the health care system and hospital practice conditions, my patience is often put to the test and strained; an impatient and rushed physician is the opposite of empathic.” (internal medicine, male)

Only one participant mentioned that having enough time had helped her develop or maintain empathy.

Bad time management and other aspects of workplace organization and structure were perceived as negative, too, for example: 

“Time taken up by tasks that are far away from the patient (e.g., documentation paperwork) is not available for deepening empathetic approaches.” (psychosomatic medicine, male)

“The medical health care system in Germany hardly allows it.” (pediatrics, male)

## Discussion

This study explored physicians’ perceptions of what promoted and inhibited their empathy development, and what was influential in the course of their training and medical education. The respondents to our survey mentioned various factors related to the medical curriculum, including some of its subjects, regular contact with patients, interactions with other physicians, their own reflection and active self-development, and workplace organization and structure. We have summarized these main themes with specific examples in Figure 1.

From these results, we reach two hypothetical conclusions: 

1. The “complex, multi-dimensional concept” of empathy [1] is influenced by the similarly complex and broad range of factors described, and some of these factors are interrelated.

2. A number of factors can be a starting point for the promotion of clinical empathy.

In the following subsections, we further elaborate on these conclusions.

### Physician empathy in medical education and practice

#### ***A lack of empathy education in the formal curriculum***

Medical education should aim to develop clinical empathy and professionalism [1,3-6,23,24,26-28]. However, our results show that this might not be happening, at least not explicitly. A substantial number of our respondents, 14 of 42, described a lack of positive influences, and one summarized it this way: “empathy was not included in medical education” (male, pediatrics).

Some physicians did mention positive aspects of the formal medical curriculum. Yet these were mainly located “on the fringe” of the curriculum, addressed as ways to teach communication between physician and patient and including a discussion of psycho-social issues. Examples are communication and attentiveness training, medical ethics, complementary and alternative medicine, and training in psychology/psychiatry/psychotherapy. Of the core medical subjects, general practice and palliative care were mentioned as positive influences on the development of empathy. The respondents did not mention other disciplines—e.g., internal medicine, surgery, or pediatrics—as either positive or negative, although we had asked them to consider any aspect of their medical education.

Qualitative research with medical students on factors influencing their empathy also found that some “fringe subjects” provided a positive influence—e.g., “classes on empathy and ethics” ([36], personal communication from the author), the behavioral sciences and medical humanities [37], and other subjects [35]. Quantitative studies confirm the positive role of training in communication skills [10,32,51] and show that a positive attitude towards psycho-social aspects of care are associated with more empathetic behavior [52]. These findings leave the impression that medical education does not currently pay enough attention to the development of clinical empathy or to subjects that promote it in the formal curriculum. This view is supported by the fact that medical students tend to perceive psycho-social aspects of care as less important than the main, formally graded subjects [39].

Although we explicitly asked respondents to think about the content of their formally endorsed medical curricula, the majority of their statements were about aspects of the working and learning environment and organizational culture [53], and about extracurricular activity. Physicians responded in a similar way to another question in our questionnaire, “What barriers to behaving in an empathetic manner do you experience in your daily professional life?” They referred to the workplace and organizational environment, patient characteristics—e.g., “difficult patients”— and their own personal attitudes as barriers to showing empathy to patients [38]. This might indicate that empathy is influenced far more by these latter aspects than by the formal curriculum, as has been argued elsewhere [6,29]. Also, the fact that respondents had taken the initiative to pursue reflective practice and self-development and saw those activities as positive influences seems to indicate that these contribute to the development of empathy but are lacking in current curricula.

#### ***The influence of role models, practice-based training, and patient contact***

A prominent aspect of the learning environment is the influence of role models [2,6] who provide both positive and negative influences on the development of empathy (see Table 2). In the perception of medical students, the role modeling of empathy provided by physicians was a central influence [29,33-37], and it has been discussed as one factor influencing the development of empathy [29]. Students viewed faculty who were empathetic towards them as influential in the students’ own development of empathy [2,35]. Teachers in primary care share this view [19], which is mirrored by our respondents’ comments that teamwork and constructive professional exchange promoted empathy development and hierarchical structures inhibited it.

Another factor that touches on aspects of the “informal” and the “hidden curriculum” [53] is contact with patients, together with practice-based training. While most of our respondents described both of these as helpful for the development of empathy, and students have done so in some studies [35,36], other investigations have reported a both positive and negative role of practice experience [33,38] or even a predominantly detrimental effect: Two of this article’s authors and other colleagues showed in the review mentioned earlier that self-assessed empathy dropped statistically significantly after students began the clinical phase of training—i.e., after contact with patients had started or intensified [29]. They suggested that the decline in empathy might be an internal reaction against overwhelming exposure to sickness, suffering, and death on the one hand and growing responsibilities on the other. Burks and Kobus have described a similar mechanism [2]. One possible explanation of the different perceptions of the impact of clinical practice is that our respondents have learned how to acquire and maintain empathy, while students have not yet done so. Most of our respondents were middle-aged (see Table 1), so this could well be the case. Another explanation is that factors other than patient contact itself cause a decline in empathy during clinical practice training—for example, the lack of opportunities for reflection on clinical experiences, or interaction with patients under stressful circumstances and time pressure.

#### ***Physicians’ stress, well-being, and focus of attention***

Stress has been identified as a significant factor in the decline in empathy during medical school and residency [29]. Neurobiology shows that the neuronal basis for empathy, mirror neurons, stop working in the presence of stress, fear, and tension [5].

On the one hand, in our study, some respondents mentioned factors that might cause such stress. For example, regarding working conditions, which respondents perceived rather negatively, the most prominent ones cited were lack of time and time pressure. These perceptions are in line with the students’, who also experienced time constraints as inhibiting empathy [33-37]. In addition, another study suggested that time pressure is a prominent barrier to physician empathy and that organizational changes could reduce this effect [11]. Other possible causes of stress mentioned in the present investigation were pressure to perform, rivalry, exhaustion, night duty, overwork, and over-fatigue. Taking social conditions among physicians into account, such as teamwork vs. hierarchy as mentioned above, our results are in line with medico-sociological findings. These show that stress and well-being in the workplace arise from organizational, psychological, and social factors [54]. Because stress not only diminishes empathy towards patients but also affects workers’ health [11,29,54], it probably has a dual detrimental effect on health care quality.

On the other hand, our respondents perceived extracurricular activities and experiences as positive for their development of empathy, which are in part identical to factors helping physicians to stay well during their professional lives [6,55-57]. Also, they have been discussed as empathy promoters [2,6,58,59]. These included personal and guided reflection, active self-development, and non-medical experiences. Respondents also mentioned aspects of professional interaction, such as working in an interdisciplinary team and professional mutual support. Physician well-being, in turn, has been associated with greater empathy and professionalism, while stress, distress, depression, and burnout have all been associated with impaired empathy and lower-quality patient care [6,59-61]. Therefore it is possible that well-being and distress are major determinants of physician empathy—but also that the presence or lack of empathy can be a determinant of physician well-being and stress.

Some aspects of medical practice were mentioned as negative factors in the development of empathy. Physicians stated that the “pure focus on … diagnostics and therapy” (pediatrics, male), “too strict adherence to guidelines” (pediatrics, male), and the reduction of pain and suffering to scientific phenomena were negative influences. In contrast, education fostering a focus on physician-patient interaction and psycho-social aspects of care were described as promoting empathy. Could an *exclusive* focus on abstract facts rather than on specific patients be one way to lose an empathic connection to them? The evidence from our study alone is weak. However, neurobiological research has shown that “attention processes affect the level of empathy, with distraction reducing it” [62]. And a recent study indicates that physicians can improve empathic interactions with patients by focusing consciously, with curiosity, and with openness on the present encounter [58]. Medical students’ focusing and concentration on one patient was positively correlated with empathy in another study [21]. This could mean that empathy is not only something that *just happens* but also something that requires *concentration* and *determination.*

### Limitations and strengths

There are a number of limitations to our study. First, the written, semi-standardized survey did not allow for the interpretation of hidden meanings, which would have required more in-depth data collection and analysis. Interviews would likely have produced more complex and nuanced results because they would have allowed us to explore the relationship between medical education and its impact on physician empathy in greater depth. However, the data produced by interviews would have exceeded the exploratory character of our study. For those reasons, our findings cannot be the basis for theory-building but instead produced hypothetical conclusions [40].

Also, our survey question was based on the assumption that physician empathy is influenced by specific elements during medical training in either a positive or a negative way. This limitation might have excluded other important influences on our respondents’ empathy. Still, many participants expanded in their responses on the factors they believed influenced the development of empathy.

A third limitation is the fact that our sample was generated from the researchers’ network and the respondents represented only four disciplines. On the one hand, this sampling procedure might have generated a generally positive self-selection bias towards clinical empathy and excluded vital experiences of other types of physicians.

On the other hand, it elicited responses from physicians in both in-patient and out-patient institutions, and it achieved a good response rate of 67%, which certainly broadened the scope of our study.

A second advantage was collecting the data anonymously; anonymity encourages respondents to answer questions honestly, rather than in ways that are deemed “socially acceptable” [42].

Third, our study seems to be the first that explicitly asked physicians for their view on the determinants of empathy development during their medical education. In addition, while many studies have treated physician empathy as completely separate from the biomedical side of medicine, or have focused on the influence of the “informal” or “hidden curriculum,” or on negative influences within the formally endorsed medical curricula [30,53], we included these relations through the openness of our qualitative design.

Finally, our results show parallels with existing research, and our hypothetical conclusions are valuable as an empirical basis for future research and possible implications for practice.

### Implications for practice and research

In what ways might our conclusions be incorporated into ideas about medical training in order to improve physician empathy? Certainly the elements of the curriculum that are intended to foster good physician-patient interaction and to include psycho-social aspects of care should be used more extensively. (A complete list of those elements mentioned in our study is included in the coding scheme in the Additional file 1: Appendix.) Also, communication skills training seems to be effective in providing patient-perceived [10,63] and observer-perceived aspects of empathy [64]. Another important approach could be to include reflective practice lessons and self-development opportunities into the medical curriculum, which some respondents had pursued as extracurricular activities that fostered their empathy.

However, when considering specific interventions to enhance empathy development, it might be wise to include learners’ individual needs in such approaches and base them on experiential learning [1,2]. For example, the hospitalization of healthy medical students has been found to have a positive effect on their empathy, as has being a patient companion during a hospital stay; both experiences emphasize the importance of patient contact [32]. A “hands-on workshop” in clinical empathy can include reflection and creative elements such as photography and role play as well as theoretical background when required. The workshop concept is based on the assumption that each health care provider develops an individual concept of empathy during the course of their lives so that the application of empathy as an interpersonal skill is the result of each actor’s experiences. The effect of such an intervention on the development of empathy measures, however, is yet to be investigated [65].

Practice-based training is very likely to have an important influence on the development of empathy, too. Special attention could be given to longitudinal care for the chronically ill and the disabled [66,67], in order to establish better relations with patients that help to develop empathy [19]. For students, practice experiences expose them to potential role models; for physicians, exposure to role models is ensured through collaboration with colleagues in their daily practice. In this context, it would seem valuable to raise physicians’ awareness that they are role models for each other and that they actively shape the organizational culture by the way they interact [68]. Because the circumstances of clinical practice might either promote or hinder the development of empathy, as was explained earlier, it is important to emphasize guidance and supervision in a clinical setting, which can be realized in a number of ways. For example, Balint groups, “meaningful experiences and reflective practice discussions”, and reflective writing sessions have been reported to help physicians deal with their experiences [5,29,69].

Such approaches, together with self-awareness training, the especially well-researched mindfulness-based stress reduction, and coaching/mentoring on a personal level could also be promoted to reduce stress and its detrimental effect on physician empathy [2,5,6,29,58,67,70]. For stress reduction, and from an organizational-structural point of view, special attention should be given to involvement in decision-making processes and the individual physician’s control over his or her practice [54]. Organizational culture and the social environment also influence stress levels. Leaders, especially, are in a position to create teamwork and mutual appreciation and support. However, “everyone in an organization … can foster a healthy organizational culture by thoughtful attention to communication, relationships, self-awareness, and the … significance of policies and behaviors” [54,68].

There is still a need for more empirical research to define the ways in which empathy can be promoted and how we can create a “culture of care” [6]. Both theory-based quantitative investigations and in-depth qualitative methods can be used, and one task for future investigations is to validate or disprove our hypothetical conclusions. However, we would like to repeat the call for more extensive use of qualitative research [31,33], because the subject is still “under researched” [31], especially from the “point of view of the person[s] concerned” ([71], p. 17).

## Conclusion

This study is an exploration of the physician’s perspective on the determinants of clinical empathy during medical education. Our most important finding is that influences on the development of physician empathy probably include a wide field of factors—curricular, social, organizational, and individual. Our results identify main themes and manageable practices that seem to influence empathy. These are good starting points for the development of theories, and for the investigation of sound interventions to improve physician and medical student empathy. The ultimate goal is better quality of care and quality of life for both physicians and patients.

## Competing interests

The authors declare that they have no competing interests.

## Authors’ contributions

FA helped to transcribe the responses, analyzed them, developed the hypothetical conclusions, and wrote the manuscript. MN and CS planned and conducted the study and supervised FA in his research. HG revised the manuscript critically for important intellectual and methodological content. EGH was involved in the counseling of FA on medical education issues, and revised the manuscript for important intellectual content. All authors reviewed the manuscript and approved of the final version.

## Authors’ information

This publication is part of FA’s medical dissertation on medical education and clinical empathy.

## Pre-publication history

The pre-publication history for this paper can be accessed here:

http://www.biomedcentral.com/1472-6920/14/122/prepub

## Supplementary Material

Additional file 1 **Appendix. **The Appendix contains a translation of the questionnaire we administered, the original questionnaire in German, more quotations from our survey, the original quotations in German, and the coding scheme we used. Click here for file
