# Supplementary material for: Determinants of physician empathy during medical education: hypothetical conclusions from an exploratory qualitative survey of practicing physicians
Source: BMC Med Educ. 2014 Jun 22;14:122. doi: 10.1186/1472-6920-14-122 (PMC4080581; doi:10.1186/1472-6920-14-122)
Supplement: Additional file 1 — Appendix. The Appendix contains a translation of the questionnaire we administered, the original questionnaire in German, more quotations from our survey, the original quotations in German, and the coding scheme we used. [file 1472-6920-14-122-S1.pdf]

Supplementary material to the research article: "Determinants of physician empathy during medical education: Hypothetical conclusions from an exploratory qualitative survey of practicing physicians," published in BMC Med Educ, Forthcoming 2014.

Appendix

Florian Ahrweiler* Melanie Neumann
Hadass Goldblatt Eckhart G. Hahn
Christian Scheffer

April 6, 2014

*Corresponding author. E-mail: florian.ahrweiler@uni-wh.de. For authors' affiliations, please see the main article.

Contents

A. Qualitative Short Survey questionnaire **3**

B. Qualitative Kurzumfrage—German original version of the questionnaire **8**

C. More physician statements **13**

D. German original physician statements **15**

D.1. Main article 15

D.1.1. Results section 15

D.1.2. Discussion section 18

D.2. Additional quotations included in this document 18

E. Coding scheme **21**

A. Qualitative Short Survey questionnaire

On the following pages you will find the translations of the questionnaire and cover letter we sent to our participants.

Short survey

Empathy in daily medical practice!?

Dear Respondent,

The working group **“Development and promotion of physician empathy in medical education and patient care”** of the Medical Faculty of Witten/Herdecke private University is conducting a brief survey.

We want to know how you personally experience empathy towards patients in your daily medical practice and how you think about it.

This survey is the first to be conducted in Germany on this topic. And internationally, there have been very few studies of the views of practicing physicians. The results will be published this year in a special issue of the journal *Rehabilitation* [*Die Rehabilitation*] entitled “Communication between patients and practitioners in medical rehabilitation.”

Please complete the attached questionnaire in its entirety and return it to us in the enclosed self-addressed envelope or by fax: 02330-62-4062.

Your data are subject to **§ 3 paragraph 6 of the federal data protection act**. This means that this survey is completely anonymous and none of your answers can be connected to you personally at any time. Therefore please do not give either your name or your address! Your answers will be translated into numbers and stored electronically. The scientific evaluation will be conducted by the study's research team: Dr. rer. medic. Melanie Neumann, Dr. med. Friedrich Edelhäuser, Dr. med. Christian Scheffer, Dr. med. Alfred Längler, Diethard Tauschel, and Dr. med. Gabriele Lutz.

If you have questions, you are welcome to contact Dr. Melanie Neumann on weekdays from 9 a.m. – 5 p.m. at 02330-62-3967, or by email at melanie.neumann@uni-wh.de. She will be happy to return your call or respond to your message.

Thank you for your time and your confidence in our scientific work!

Results of the research

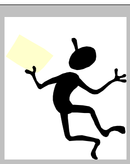

Dear Respondent,

If you are interested in the results of this survey, we will be pleased to send you the aforementioned scientific publication free of charge by post or email. To request a copy, please call Dr. Neumann at 02330-62-3967 or send an email to melanie.neumann@uni-wh.de with your name, postal address and email address.

1. What do you understand by empathy in medical practice?

2. What educational content in the course of your studies and/or your specialist training had a positive or negative effect on your empathy?

positive:

negative:

Please be sure to answere ALL of the questions!

3. In what ways can an empathic doctor influence the health and convalescence of the patient?

4. What barriers to behaving in an empathetic manner do you experience in your daily professional life?

Please be sure to answer ALL of the questions!

5. In what situations do you succeed in behaving in a particularly empathetic manner towards your patients?

____

____

____

____

____

____

____

____

____

____

____

____

____

6. Please rate the following statements.

Please provide **only one answer per line!**

**Medical empathy improves:**	strongly disagree	somewhat disagree	somewhat agree	strongly agree
communication with the patient	☐	☐	☐	☐
anamnesis (taking the medical history)	☐	☐	☐	☐
diagnosis	☐	☐	☐	☐
the giving of information to the patient	☐	☐	☐	☐
the education of the patient	☐	☐	☐	☐
therapy	☐	☐	☐	☐
physical therapeutic outcome	☐	☐	☐	☐
psychosocial therapeutic outcome	☐	☐	☐	☐

7. Finally, please provide a few personal details about yourself.

age: ____________ years male ☐ female ☐

medical specialization: ____

number of years in practice _____________ as a medical specialist ☐ as a medical assistant ☐

Employed in a doctor's office for _______________ years Employed in a hospital for _______________ years

University hospital ☐ No university hospital ☐ Hospital for complementary medicine ☐

Please be sure to answer ALL of the questions!

B. Qualitative Kurzumfrage—German original version of the questionnaire

The German originals of the questionnaire and cover letter used in our survey are presented on the following pages.

Kurzumfrage

Empathie im ärztlichen Alltag!?

Sehr geehrte Befragte/ sehr geehrter Befragter,

die Arbeitsgruppe **„Entwicklung und Förderung ärztlicher Empathie in Ausbildung und Patientenversorgung“** der Medizinischen Fakultät der Privaten Universität Witten/Herdecke führt eine Kurzumfrage durch.

Uns interessiert, wie Sie persönlich Empathie gegenüber Patienten im ärztlichen Alltag erleben und darüber denken.

Diese Umfrage ist die erste in Deutschland und auch international liegen bisher kaum Untersuchungen zur Sichtweise praktisch tätiger Ärzte vor. Die Ergebnisse werden im Schwerpunktheft „Die Kommunikation zwischen PatientInnen und BehandlerInnen in der medizinischen Rehabilitation" in der Zeitschrift „Die Rehabilitation“ in diesem Jahr veröffentlicht.

Bitte füllen Sie den beiliegenden Fragebogen vollständig aus und senden ihn dann im bereits adressierten freien Antwortkuvert oder per Fax 02330-62-4062 an uns zurück.

Ihre Angaben unterliegen **§ 3 Absatz 6 des Bundesdatenschutzgesetzes**. Das bedeutet, dass diese Befragung völlig **anonym** ist und deshalb auch zu keinem Zeitpunkt Rückschlüsse auf Ihre Person möglich sind. Geben Sie daher bitte auf diesem Fragebogen weder Ihren Namen noch Ihre Adresse an! Ihre Antworten werden in Zahlen umgesetzt und auf einem elektronischen Datenträger gespeichert. Die wissenschaftliche Auswertung erfolgt ausschließlich durch das Forscherteam: Dr. rer. medic. Melanie Neumann, Dr. med. Friedrich Edelhäuser, Dr. med. Christian Scheffer, Dr. med. Alfred Längler, Diethard Tauschel, und Dr. med. Gabriele Lutz.

Sollten Sie Rückfragen haben, können Sie sich gerne werktags von 9.00-17.00 Uhr unter der Telefonnummer 02330-62-3967 oder per Email melanie.neumann@uni-wh.de an Frau Dr. Melanie Neumann wenden. Sie ruft Sie zur Beantwortung Ihrer Fragen auch gerne zurück.

Wir bedanken uns für Ihre Zeit und Ihr Vertrauen in unsere wissenschaftliche Arbeit!

Ergebnisse der Studie

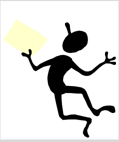

Sehr geehrte Befragte/ sehr geehrter Befragter,

wenn Sie Interesse an den Ergebnissen dieser Studie haben sollten, schicken wir Ihnen gerne die o.g. wissenschaftliche Veröffentlichung **kostenfrei per Post oder Email** zu. Für diesen Fall rufen Sie Frau Dr. Neumann unter 02330-62-3967 an oder schreiben Sie Ihr eine Email melanie.neumann@uni-wh.de und teilen Sie Ihren Namen sowie Ihre Anschrift oder Email mit.

1. Was verstehen Sie unter ärztlicher Empathie?

2. Durch welche Ausbildungsinhalte in Ihrem Studium und/oder in Ihrer Facharztausbildung wurde Ihre Empathie sowohl positiv als auch negativ beeinflusst?

positiv:

negativ:

Bitte prüfen Sie, ob Sie **ALLE** Fragen **VOLLSTÄNDIG** beantwortet haben!

3. Auf welche Art und Weise kann ein empathischer Arzt die Gesundheit und Genesung des Patienten beeinflussen?

4. Welche Barrieren erleben Sie in Ihrem Berufsalltag sich empathisch gegenüber Ihren Patienten zu verhalten?

Bitte prüfen Sie, ob Sie **ALLE** Fragen **VOLLSTÄNDIG** beantwortet haben!

5. In welchen Situationen gelingt es Ihnen sich besonders empathisch gegenüber Ihren Patienten zu verhalten?

____
____
____
____
____
____
____
____
____
____
____

6. Bitte beurteilen Sie die folgenden Aussagen.

Bitte kreuzen Sie in **jeder Zeile nur eine Antwortmöglichkeit** an!

**Ärztliche Empathie verbessert:**	stimme überhaupt nicht zu	stimme eher nicht zu	stimme eher zu	stimme voll und ganz zu
Kommunikation mit dem Patienten	☐	☐	☐	☐
Anamnese	☐	☐	☐	☐
Diagnose	☐	☐	☐	☐
Information des Patienten	☐	☐	☐	☐
Patientenedukation	☐	☐	☐	☐
Therapie	☐	☐	☐	☐
Physischen Behandlungserfolg	☐	☐	☐	☐
Psychosozialen Behandlungserfolg	☐	☐	☐	☐

7. Zum Abschluss bitten wir Sie noch um einige Angaben zu Ihrer Person.

Alter: ___________ Jahre　　männlich ☐　　weiblich ☐

Medizinische Fachrichtung: ______________________________

Jahre im Beruf ____________　　Facharzt ☐　　Assistenzarzt ☐

Tätig in Praxis seit _______________ Jahren　　Tätig in Klinik seit _______________ Jahren

Uniklinik ☐　　Keine Uniklinik ☐　　Komplementärmed. Klinik ☐

Bitte prüfen Sie, ob Sie **ALLE** Fragen **VOLLSTÄNDIG** beantwortet haben!

C. More physician statements

The additional statements provided in this section offer further evidence of the themes presented and discussed in the main article.

> "Is empathy something you learn during your medical education or is it something you already possess!?!?"
> (internal medicine, male)
>
> "positive: good clinical instructors[;] clinical courses[;] excellent training during the medical internship in the UK[;] clinical electives in anthroposophical clinics/doctor's offices
> negative: function ... [illegible] hierarchical structures[;] stress about work, overwork[;] 'The cool statistical perspective[?, illegible] of evidence-based medicine'[;] un-empathetic senior physicians[;] un-empathetic colleagues"
> (internal medicine, male)
>
> "positive: from observation and then investigation of possible suspected diagnoses"
> (rehabilitation medicine, male)
>
> "positive: I experienced only positive effects. For example, from training in medical ethics and psychology, and in daily practice"
> (surgery, male)
>
> "positive: good teamwork, professionally skilled colleagues, and well-trained doctors[;] cooperation with specialists in other disciplines
> negative: increasing bureaucracy[;] deterioration in the hospital's organization[;] an insufficient number of professional workers in social services and the typing service"
> (internal medicine, male)
>
> "positive: contact with patients, training in taking a patient's medical history
> (supervised and with an interview partner), raising the topic of salutogenesis
> negative: I can't really say; it could even be said that 'non-empathetic' behaviors also provide some positive training"
> (internal medicine, female)
>
> "positive:
>
> - encounters with empathetic doctors

- presentation of patients' cases within the frameworks of a palliative care panel, of a conference with therapists and physicians, or a complementary medical case conference
- practical training on a palliative care unit

negative:

- encounter with emotionally reserved or detached doctors
- hierarchical structures instead of teamwork on eye-level"

(internal medicine, male)

"In my opinion, it is less the concrete content [that promotes empathy,] than the personal skills of every physician, which certainly can be trained, too."
(pediatrics, male)

"positive: In the course of my studies there were no influences. During medical training, [there were positive influences] from further inquiries by the instructors or by what the patient wanted to communicate.
negative: Because of the large number of patients, especially in providing emergency services, it was all about a fast result."
(pediatrics, male)

"Closer analysis of the children's and parents' biographies, first in connection with chronic diseases, later in 'acute' situations. 'Mentors' were mostly families, but also two brilliant principals (of 5 chief physicians)
negative: focusing only on clinical diagnosis and therapy, idiotic time management"
(pediatrics, male)

"positive: student exercises in obtaining the medical history, and integrating medical evidence, the medical condition, and psychosocial circumstances
negative: too much emphasis on pure factual knowledge, e.g., in preparation for exams"
(pediatrics, male)

"contact with human beings (not bodies or 'patients')"
(general medicine, male)

"positive: specialist training: not the explicit training, but the exemplary behavior of colleagues, which inspired us to follow their

example”
(internal medicine, male)

“I cannot remember any aspect of my medical education that dealt with empathy”
(rehabilitation medicine, male)

“positive: During the specialist training empathy was exemplified by some trainers; in some education courses and through an exchange of ideas with colleagues, I was able to benefit from their experience. negative: In the course of my studies, I can only remember empathy being defined as a concept.”
(general medicine, female)

“positive: Balint group during my studies, during the medical internship, and in the following years, also after completion of the advanced training”
(general medicine, male)

D. German original physician statements

This section contains the original German statements by our respondents. These are presented in translation in the main article and in section C on page 13 of this document.

D.1. Main article

D.1.1. Results section

In general, medical education does not promote the development of empathy

„ausschließlich (beinahe) Eigeninitiative, learning by doing“
(Innere Medizin, männlich)

„Es gab keine Ausbildungsinhalte in Studium und Facharztausbildung, die Empathie überhaupt berücksichtigen.“
(keine Details angegeben)

„Es gab keine expliziten noch implizite Ausbildungsinhalte, die mich gelehrt hätten, dass Empathie nicht erstrebenswert wäre.“
(Innere Medizin, männlich)

Recognizing the psycho-social dimensions of care fosters empathy

„Ausbildung in Gesprächsführung!!!“
(Allgemeinmedizin, weiblich)

„Ausbildung in Psychiatrie und Psychotherapie“
(Neurologie und Psychiatrie, weiblich)

„Fächer Medizinische Ethik, Psychologie“
(Chirurgie, männlich)

„Im Laufe meines Studiums nahm ich an einem ‚Wahrnehmungskurs‘ teil, welcher auch das Thema Projektion behandelte, dies hat mir den Blick dafür geöffnet.“
(Innere Medizin, weiblich)

„Zahlreiche Leitlinien und deren ‚Abarbeiten‘ empfinde ich als kontraproduktiv (innere Agenda ist eher leitlininengerichtet als patientenorientiert)“
(Allgemeinmedizin, männlich)

„Leiden/Schmerz wurden auf naturwissenschaftliche Phänomene reduziert, die pharmakologisch angehbar waren.“
(Pädiatrie, männlich)

Interactions with patients in medical practice promote empathy

“in der Ausbildung ... was der Patient mitteilen wollte”
(Pädiatrie, männlich)

“kritische Rückmeldungen der Patienten”
(Pädiatrie, weiblich)

Physicians’ active self-development through reflective practice helps the development of empathy

“ausschließlich (beinahe) Eigeninitiative”
(Innere Medizin, männlich)

“selbstorganisierte studentische Anamnesegruppe[,] studentischer Selbstuntersuchungskurs[,] Gesprächsführungskurs Psychosomatik[,] Balint-Gruppe[,] Selbsterfahrungsgruppe in Kinder- und Jugend-Psychiatrischer Weiterbildung[,] ... Projektleiter der AG Kinderklinik im Projekt Tod und Sterben im [Name des Krankenhauses]”
(Pädiatrie, männlich)

“Supervision und Weiterentwicklung der eigenen Persönlichkeit”
(Neurologie und Psychiatrie, weiblich)

“Begegnungen mit lebenserfahrenen, weisen Persönlichkeiten”
(Pädiatrie, männlich)

“Studium generale[;] Mitteilnahme an anderen Vorlesungen, zum Beispiel Psychologie”
(keine Details angegeben)

“Meine Empathie beruht eher auf meinem christlichen Menschenbild.”
(Rehabilitationsmedizin, männlich)

Interactions with colleagues can both promote and inhibit empathy through their role modeling of empathic and non-empathic behavior

„erfahrene Kollegen, die eine empathische Umgangsweise mit Patienten pflegen“
(Pädiatrie, männlich)

„Während Studium und Ausbildung wurde häufig darauf hingewiesen ‚Versuchen Sie sich in die Situation des Patienten zu versetzen.‘ “
(Rehabilitationsmedizin, männlich)

„Beispiele für Empathie waren eher Kollegen/Vorgesetzte, die sowohl positive als auch negative Beispiele gaben.“
(Rehabilitationsmedizin, männlich)

„eher dadurch, dass viele andere Kollegen (z. B. auch universitäre Schulmediziner) nicht ausreichend auf ihre Patienten und deren Probleme eingehen. (Stichwort: ärztliche Arroganz, ‚Götter in Weiß‘)“
(Innere Medizin, männlich)

„Zynismus und Patienten als Futter für ‚Studien‘ “
(Allgemeinmedizin, männlich)

„hierarchische Strukturen statt Teamwork auf gleicher Augenhöhe“
(Innere Medizin, männlich)

„schlechte Ausbilder“
(Innere Medizin, männlich)

Stress, time pressure, and adverse working conditions are detrimental to empathy development

> „für Prüfungen zu ‚pauken'"
> (Pädiatrie, männlich)

> „durch den allgemeinen Krankenhausstress"
> (Pädiatrie, männlich)

> „Häufig [ist es] durch Zeitdruck und [die] Anzahl von Patienten kaum möglich, sich empathisch auf Eltern und Patienten einzustellen."
> (Pädiatrie, männlich)

> „Durch Zeitdruck aufgrund der Umstände im Gesundheitssystem/Krankenhausalltag wird meine Geduld oft auf die Probe gestellt und strapaziert; ein ungeduldiger und gehetzter Arzt ist das Gegenteil von Empathie."
> (Innere Medizin, männlich)

> „Negativzeiten, die durch patientenferne Tätigkeiten in Anspruch genommen werden (zum Beispiel Dokumentation), stehen für die Vertiefung empathischen Vorgehens nicht zur Verfügung"
> (Psychosomatische Medizin, männlich)

> „medizinisches Gesundheitssystem in Deutschland erlaubt es kaum"
> (Pädiatrie, männlich)

D.1.2. Discussion section

> „Empathie stellte keinen Ausbildungsinhalt dar."
> (männlich, Pädiatrie)

> „Reines Fokussieren auf die ... Diagnostik und Therapie"
> (Pädiatrie, männlich)

> „zu starres Festhalten an Leitlinien"
> (Pädiatrie, männlich)

D.2. Additional quotations included in this document

The English translations can be found in section C on page 13.

> „Lernt man Empathie in der Ausbildung oder bringt man sie mit!?!?"
> (Innere Medizin, männlich)

„positiv: gute klinische Lehrer[;] klinische Kurse[;] exzellente Ausbildung im Praktischen Jahr im UK[;] Famulaturen in anthroposophischen Klinik[en]/Praxen
negativ: Funktion . . . [unleserlich] hierarchische Strukturen[;] Arbeitsüberlastung, Überforderungen; ‚Der kühle statistische [?, unleserlich] der Evidenz-basierten Medizin'[;] un-empathische Oberärzte[;] unempathische Kollegen"
(Innere Medizin, männlich)

„positiv: Durch Verlaufsbeobachtungen und somit Aufklärung etwaiger Verdachtsdiagnosen"
(Rehabilitation, männlich)

„positiv: Ich hatte nur positive Einflüsse erlebt. Z.B. durch die Fächer medizinische Ethik, Psychologie und in der täglichen Praxis"
(Chirurgie, männlich)

„positiv: Gute Teamarbeit, fachkompetente Kollegen und gut ausgebildete Ärzte[;] Zusammenarbeit mit anderen Fachdisziplinen[;]
negativ: zunehmende Bürokratie; Verschlechterung der Organisation im Krankenhaus; nicht ausreichende Fachkräfte im Sozialdienst und Schreibdienst"
(Innere Medizin, männlich)

„positiv: Patientenkontakte, Anamnesetraining (supervisiert und mit Gesprächspartner), Thematisierung von Salutogenese
negativ: kann ich nicht sagen; auch ‚nicht empathische' Situation zu bemerken, schult ja eher positiv im Hinblick auf empathisches Verhalten"
(Innere Medizin, weiblich)

„positiv:

- Begegnung mit empathischen Ärzten
- Patientenvorstellungen im Rahmen eines palliativen Gremiums, einer Besprechung von Therapeuten und Ärzten oder einer komplementär-medizinischen Fallbesprechung
- Praktikum auf einer Palliativstation

negativ:

- Begegnung mit distanzierten Ärzten.
- Hierarchische Strukturen statt Teamwork auf gleicher Augenhöhe"

(Innere Medizin, männlich)

„Es sind meines Erachtens weniger konkrete Inhalte, als die persönlichen menschlichen Fähigkeiten jedes Arztes, die natürlich auch geschult werden können."
(Pädiatrie, männlich)

„positiv: Im Studium fand keine Beeinflussung statt. In der Ausbildung durch Rückfragen der Ausbilder/was der Patient mitteilen wollte
negativ: Durch die Vielzahl der Patienten, insbesondere in Notdiensten ging es nur um ein schnelles Resultat"
(Pädiatrie, männlich)

„positiv: Auseinandersetzung mit der Biografie der Kinder und Eltern, zunächst im Begleiten chronischer Erkrankungen, später konnte ich diese Aspekte auch in der ‚Akuten' Situation erfahren. ‚Lehrmeister' waren überwiegend die Familien, aber auch zwei geniale Chefs (von 5 Chefärzten insgesamt)
negativ: Reines Fokussieren auf die klinische Diagnostik und Therapie, blödsinniges Zeitmanagement"
(Pädiatrie, männlich)

„positiv: Studentische Übungen, Anamnese zu erheben, Befunde, Befinden und psychosoziale Umstände zu integrieren
negativ: Zu viel reines Faktenwissen, z.B. für Prüfungen zu ‚pauken' "
(Pädiatrie, männlich)

„positiv: Kontakt mit Menschen (nicht Leichen oder ‚Patienten')"
(Allgemeinmedizin, männlich)

„positiv: Facharzt-Ausbildung: Keine explizite Ausbildung, jedoch vorbildliches Verhalten von Kollegen, das zur Nachahmung einlud."
(Innere Medizin, männlich)

„Ich kann mich an keine Ausbildungsinhalte erinnern, die sich mit Empathie beschäftigt hätten."
(Rehabilitationsmedizin, männlich)

„positiv: In der Facharztausbildung wurde mir Empathie durch einige Ausbilder vorgelebt, in einigen Fortbildungen und im Austausch mit Kollegen/innen konnte ich sicher von deren Erfahrungen profitieren.
negativ: Im Studium ist mir Empathie als reine Definition des Begriffs in Erinnerung."
(Allgemeinmedizin, weiblich)

„positiv: Balintgruppenarbeit noch im Studium, im PJ und in nach-

folgenden Jahren, auch nach Abschluss der Weiterbildung"
(Allgemeinmedizin, männlich)

E. Coding scheme

Our coding scheme is displayed on the next page.

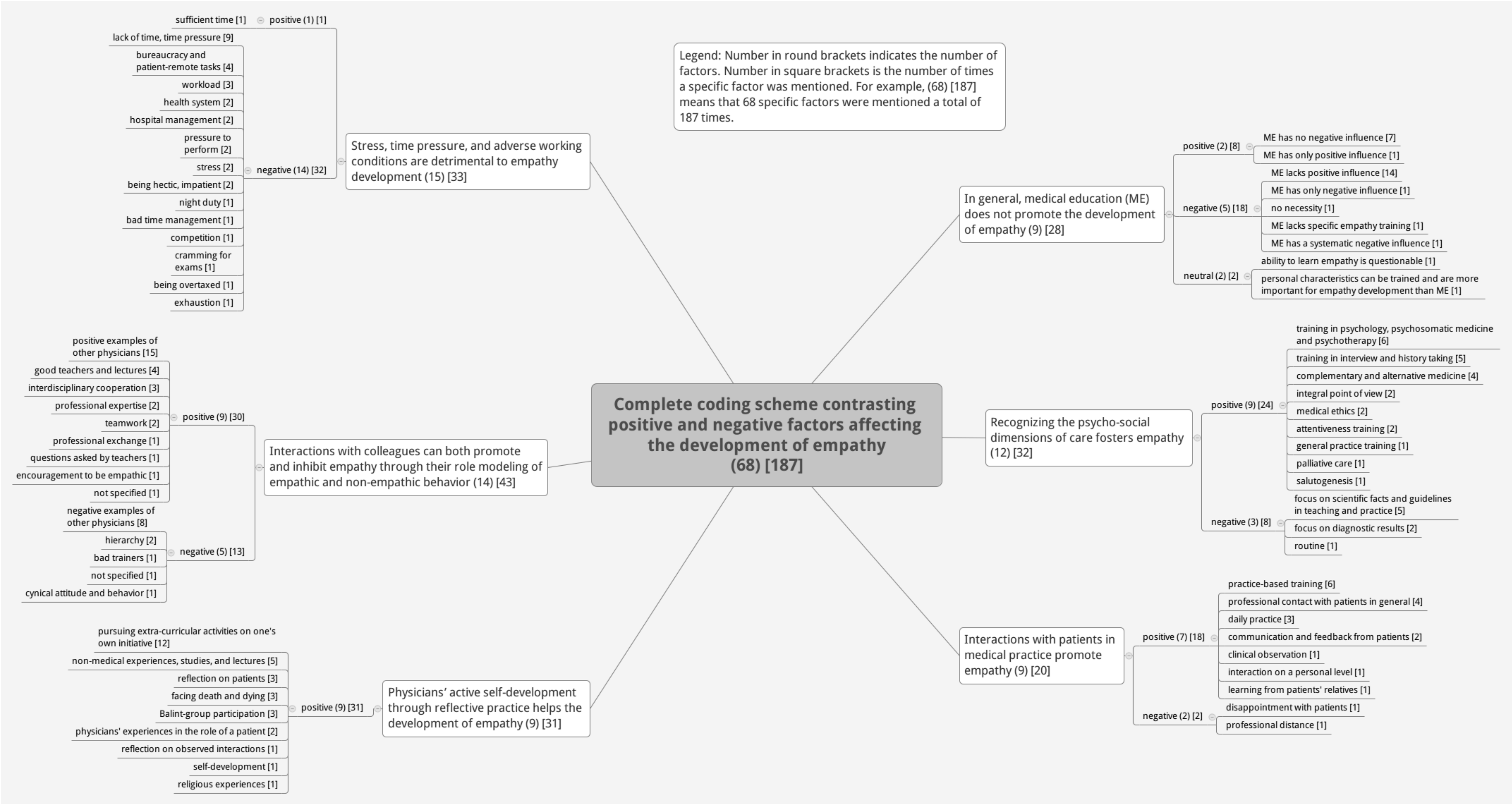
Complete coding scheme contrasting positive and negative factors affecting the development of empathy (68) [187]
Legend: Number in round brackets indicates the number of factors. Number in square brackets is the number of times a specific factor was mentioned. For example, (68) [187] means that 68 specific factors were mentioned a total of 187 times.
Stress, time pressure, and adverse working conditions are detrimental to empathy development (15) [33]
positive (1) [1]
sufficient time [1]
negative (14) [32]
lack of time, time pressure [9]
bureaucracy and patient-remote tasks [4]
workload [3]
health system [2]
hospital management [2]
pressure to perform [2]
stress [2]
being hectic, impatient [2]
night duty [1]
bad time management [1]
competition [1]
cramming for exams [1]
being overtaxed [1]
exhaustion [1]
In general, medical education (ME) does not promote the development of empathy (9) [28]
positive (2) [8]
ME has no negative influence [7]
ME has only positive influence [1]
negative (5) [18]
ME lacks positive influence [14]
ME has only negative influence [1]
no necessity [1]
ME lacks specific empathy training [1]
ME has a systematic negative influence [1]
neutral (2) [2]
ability to learn empathy is questionable [1]
personal characteristics can be trained and are more important for empathy development than ME [1]
Interactions with colleagues can both promote and inhibit empathy through their role modeling of empathic and non-empathic behavior (14) [43]
positive (9) [30]
positive examples of other physicians [15]
good teachers and lectures [4]
interdisciplinary cooperation [3]
professional expertise [2]
teamwork [2]
professional exchange [1]
questions asked by teachers [1]
encouragement to be empathic [1]
not specified [1]
negative (5) [13]
negative examples of other physicians [8]
hierarchy [2]
bad trainers [1]
not specified [1]
cynical attitude and behavior [1]
Recognizing the psycho-social dimensions of care fosters empathy (12) [32]
positive (9) [24]
training in psychology, psychosomatic medicine and psychotherapy [6]
training in interview and history taking [5]
complementary and alternative medicine [4]
integral point of view [2]
medical ethics [2]
attentiveness training [2]
general practice training [1]
palliative care [1]
salutogenesis [1]
negative (3) [8]
focus on scientific facts and guidelines in teaching and practice [5]
focus on diagnostic results [2]
routine [1]
Physicians' active self-development through reflective practice helps the development of empathy (9) [31]
positive (9) [31]
pursuing extra-curricular activities on one's own initiative [12]
non-medical experiences, studies, and lectures [5]
reflection on patients [3]
facing death and dying [3]
Balint-group participation [3]
physicians' experiences in the role of a patient [2]
reflection on observed interactions [1]
self-development [1]
religious experiences [1]
Interactions with patients in medical practice promote empathy (9) [20]
positive (7) [18]
practice-based training [6]
professional contact with patients in general [4]
daily practice [3]
communication and feedback from patients [2]
clinical observation [1]
interaction on a personal level [1]
learning from patients' relatives [1]
negative (2) [2]
disappointment with patients [1]
professional distance [1]
